# The Efficacy of the Antigonorrheic Arhovin

**Published:** 1909-02

**Authors:** 


### ABSTRACTS.

### THE EFFICACY OF THE ANTIGONORRHEIC ARHOVIN.

Professor W. Nagel, of Berlin University, from his gynecological polyclinic (*Zeitschrift f. neuere physik. Medizin,* June 1, 1908) says he has used arhovin for three years, with generally favorable results, and considers it indicated in gonorrheal diseases of the genital organs and urinary passages in the dose of two capsules thrice daily. He also employed it in the form of vaginal globules and bougies. Untoward effects from its use never occurred. The duration of the arhovin treatment is, on the average, between two and six weeks. In acute gonorrhea he considers it especially important to have the patient taking arhovin observe the dietetic and hygienic requirements. His most recent observations have certainly confirmed his earlier conclusions. The chief effects of arhovin are secretion limiting and analgesic; the discharge becomes less and the pain on urination disappears. The urine, at first cloudy, becomes clear.

